# Machine learning to predict the role of CHWs in shifting birth preferences away from homebirth in India

**DOI:** 10.1038/s41598-025-24446-1

**Published:** 2025-10-31

**Authors:** Moumita Mukherjee, Chetan Harshal Tote, Anuj Batta

**Affiliations:** 1 Charité – Universitätsmedizin Berlin, Berlin, Germany; 2 Department of Business, University of Europe for Applied Sciences, Potsdam, Germany; 3 Plenary HR, Noida, India

**Keywords:** Home birth, Community health workers, Maternal health, Machine learning, Health care, Engineering

## Abstract

This study utilized well-known supervised machine learning algorithms to NFHS‑5 data of West Bengal, India, to predict the place of birth (home vs facility) by integrating CHW (community health worker) contact factors and women participant’s perceptions about intimate partner violence (IPV). Although the study applied modelling techniques from conventional ML literature, the overarching contribution was identifying avenues to enhance public health policy response (e.g., efficient targeting of home visits and counselling by ANM/ASHA). The study concludes that, identifying likely homebirth cases among women with IPV-related poor perceptions applying improved prediction can enhance prioritising of CHW-contact and alter birth preference. The study improves minority-class learning using SMOTE on weighted NFHS data keeping in mind the complex survey design and SMOTE limitations. With respect to the ML model performance, Random Forest produced the highest test AUC (0.991) and accuracy (96.7%) among the 5 evaluated classifiers—LR (base), RF, MNB, k-NN, SVM and 0.950 with stable accuracy of 96% on hold-out data. The study does not bring methodological novelty in the underlying algorithms but generated actionable insights for equitable CHW allocation for efficient targeting using standard cross-sectional survey data.

## Introduction

Globally, the maternal mortality rate in 2020 was 103 per 100000 live births, and 16% (47000) of total maternal deaths occurred in Southeast Asia^1^. Furthermore, 2.8% of maternal deaths contribute to complications during delivery at the global level, and 2.1% occur in Southeast Asia^2^. Despite equitable achievements in access to facilities for childbirth in low- and middle-income countries (LMICs), 42% of maternal deaths and 23% of neonatal deaths occur on the first day of birth, and the place of delivery is a significant determinant of maternal/neonatal survival^3^. Equitable access to health facility birth is one primary goal of maternal and child health programmes (MCH) in LMICs like India. Access to timely, cost-effective care with the availability of all basic MCH services at health facilities reduces the chance of death from complications which are not accessible at home^3^. Identifying the influencing factors and exploring efficient solutions involving community health workers (CHWs) would accelerate progress in service delivery and ensure health seeking at facilities improving outcome^4,5^. The preference for home birth over institutional delivery in India is a crucial contributor of adverse birth outcome despite having well-planned MCH programs^6^. Ou et al.^6^ analysing National Family Health Survey (NFHS) 2015–16 data using a sample of 699686 women of reproductive age revealed that 34% of women who delivered at home perceive delivery at institutions is not necessary. On the other hand, according to the two consecutive NFHS estimates, the rate of health facility birth in the last 5 years preceding the survey increased from 79% to 89% in India, with a 12-percentage point increase in rural areas compared to a 5-percentage point increase in urban areas, indicating an increase in equitable access in rural counterparts^7^. With that said, 11% of home births are still quite high in terms of absolute numbers in a country with a population of 1.4 billion where likelihood of maternal and neonatal mortality increases with home delivery. In this context, the maternal mortality rate in India is 115 per 100000 live births, and in West Bengal, it is 109 per 100000 live births^8^. Maternal mortality in India due to obstetric complications is still significant among women where delivery takes place at home. Massive vaginal bleeding accounted for between 22.3% and 23.7% of facility-based delivery cases, which is up to 28.2% with respect to home birth cases among women aged 15 - 49 years who had given birth within the last 5 years preceding the survey^7^.

Furthermore, the risk of early neonatal mortality is greater in homebirth cases^9^ and the neonatal mortality rate (NMR) is 20 (per 1000 live births), with a 70.5% contribution of NMRs to the infant mortality rates (IMR)^7,10,11^. Though West Bengal state ranks medium in terms of human development, NMR is 14 (per 1000 live births) with higher contribution to IMR (84.2%) than national average (ibid.). Additionally, the prevalence of birth asphyxia/hypoxic ischaemic encephalopathy (19.5% in West Bengal, 15.4% in India) and sepsis (15.4% in West Bengal, 12.0% in India) are also at higher side than national estimates (ibid.). Moreover, the percentage of mothers with postnatal health check-up in the first 2 days after birth [facility birth (61.8% - 66.5%); homebirth (38.5% - 45.9%)] as per NFHS 5 findings^7^. Similar is evident in neonatal postnatal care [facility birth (86.2% - 89.8%); homebirth (21.9% - 29.8%)] – crucial service to increase survival and associated to place of delivery^7,12,13^.

With that said, evidence also shows that facility birth is a protective factor only if quality of care is ensured^14^. Based on the above analysis, it is evident that if the quality of care is improved, then delivery at institutions can increase maternal and neonatal survival. Among other quality interventions, application of artificial intelligence (AI) is ensuring quality and timely care with more accurate prediction of needs, identification of crucial determinants and started to enhance policy decision^4,15^.

### Role of access to information about pregnancy complications and birth preparedness at public facilities as major determinants

In India, the rate of home delivery is decreasing over time; however, unequal distribution of access to quality care by economic (13.1% poorest/0.8% richest), spatial (10.0% rural/4.5% urban), and social (28.6% minorities/5.0% others) factors is evident^7^. One significant contributor to lower uptake of MCH service like antenatal care is exposure to physical intimate partner violence (IPV) (aOR= 1.09, 95%)^16^, where reported prevalence of domestic violence in India from different community-based surveys is 32.0% to 77.5%^17^, 22.2% to 49.5% during pregnancy^18,19^ resulting in death during pregnancy or delivery^17,20,21^. In such a scenario, quality care during childbirth is crucial and involves not only a safe environment but also comprehensive awareness among pregnant women about birth preparedness and complication readiness^14,22^. However, lack of access to information about the availability of quality care to treat pregnancy complications, intrapartum and postpartum care, newborn care is a major barrier for making informed choice toward institutional delivery^3,23,24^ The latest National Family Health Survey revealed that contact with frontline health workers to increase knowledge about the MCH services is increasing as a part of the implementation of national health policy^7^. Therefore, information sharing by CHWs while they conduct community visits is an important step toward the safe delivery of children, access to postpartum care with the objective of improving survival outcomes in India.

Though literature shows AI applications improve CHW’s role in MCH services, gaps are visible in exploring the role of AI in strengthening CHW’s targeting of women subgroup exposed to different vulnerabilities like IPV with accurate classification. Hence, the present study aims to explore a suitable machine learning (ML) algorithm as an AI tool to make early, robust, real-time predictions of the association between information sharing of services by health workers and the choice of facility-based delivery over home delivery by training and testing five supervised learning algorithms followed by evaluations to make decisions.

### Use of machine learning algorithms as digital health tools to improve health outcomes

Digital health interventions in many forms have accelerated achievements towards Universal Health Coverage (UHC), leading to improvements in the quality of service in the dimensions of promotion, prevention, and cure^25,26^. Digitalization improves the healthcare decision-making process through the identification and prediction of needs involving CHWs through establishing direct linkages with care-seekers to ensure good-quality care^27^. Multiple studies have shown that digital health tools are increasingly effective in predicting health workers’ willingness to stay, develop perceptual abilities for basic life support, and to reduce approachability barriers^28–32^. algorithms for better predictive accuracy and precision, algorithms such as ML algorithms like random forest, support vector machine, decision tree, and k-nearest neighbor are widely used supervised learners for clinical diagnosis, prediction of program effectiveness or CHWs’ performance-based incentivisation  – supporting policy decisions^29,32^. Furthermore, some studies compare the performance of classical regression and ML models to assess the usefulness of the secondover the conventional one^5,33–37^. In addition, supervised algorithms are used to predict the risk of pregnancy and birth outcomes, such as the birth of premature neonates, the prediction of child mortality, and child anaemia, to ensure early prediction and early prevention^38–41^. Additionally, ML models help to make decisions on CHW’s retention in rural areas to assure hard-to-reach service delivery through more accurate classification of the target variable classes^32^. Among different supervised learners, random forest and k-NN are found to be the top performers in predicting onset of disease and health- seeking behaviour, easing decision making for resource allocation^5,6,36,37,42,43^.

Regarding the effectiveness of CHW-led counselling, obstetric complications such as preeclampsia, blood loss, and abnormal foetus position can be avoided with early prediction based awareness-building ensuring delivery at institutions^43–45^. A study by Rittenhouse et al.^46^ used ML algorithms to identify maternal- and newborn-level correlates and to classify preterm newborns with greater predictive accuracy. Fredriksson et al.^47^ used a random forest model to predict delivery location to ensure need-based support for women at risk of not selecting facility-based delivery in Zanzibar. In contrast, a birth registry-based cohort study in northern Tanzania compared the performance of five ML models with logistic regression found no significant difference in the prediction of perinatal deathor in identifying the key predictors^35^.

Given this context, this study includes the place of child delivery as an indicator of service availability and appropriateness. The task is to classify the choice of delivery location, identifying important features by comparing 5 supervised ML algorithms and a classical binary logistic regression model. The data used is weighted resampled data from a nationally representative dataset known as National Family Health Survey (NFHS) round 5, selecting the state of West Bengal, India. The selected ML models are random forest (RF), naïve Bayes (NB), k-nearest neighbor (k-NN), logistic regression (LR), and support vector machine (SVM)—to train and test and infer which model would be the best for the said purpose with better variance‒bias balance after running the synthetic minority oversampling technique (SMOTE) to resample the imbalanced data.

### Research questions

1. Are visits by CHWs and discussions about services related to pregnancy, childbirth, postnatal care, newborn care, and other related care positively associated with the occurrence of facility-based delivery while controlling sociodemographic and access to MCH services, maternal health and lifestyle factors?
2. Does the association vary if CHWs give special emphasis to women who perceives intimate partner violence (IPV) is justified?
3. Does the ML model perform better than the classical model in classifying the choice of home, public or private facility classes with enhanced knowledge through information sharing of health workers and special focus on vulnerable women?

### Methodology

The study analysed the Demographic and Health Survey, India - National Family Health Survey round 5 (2019 - 2021). The study selected the state of West Bengal in the eastern part of India with 5,618 observations where the observational unit is the child born to the surveyed participant woman during the last five years preceding the survey. The current study explores the role of CHWs in increasing access to facility-based birth. The analyses are carried out to explore the association in select population subgroup as well as whether it varies if a particular vulnerable women population subgroup is considered. To explore the first aspect, the final number of observations considered for analysis after data cleaning – deleting the rows with missing values – is 2918. To explore the second aspect, the vulnerable subgroup considered is a subgroup where respondents were asked the questions on the attitude of women on wife beating justification. The select subgroup was included based on criteria – women aged between 15–49 years, who are de facto residents (slept in the household the previous night), who were selected and interviewed for the Domestic Violence module after their consent and only one woman per household was selected for the Domestic Violence module, due to sensitivity. The beating justification index score is created in this study using additive principle after deleting the rows containing missing values. The index score is generated from 6 binary variables covering Justification scenario – if she goes out without telling him, if she neglects the children, if she argues with him, if she refuses sex, if she burns the food, if she doesn’t cook properly. Therefore, the score is a continuous variable with values ranging from 0 to 6. A binary variable is created for the analysis with two categories wife beating is not justified, wife beating is justified for any reason. After considering the factor women’s perception about intimate partner violence, the sample size has become 356.

To address the research questions, exploratory, and predictive analyses are conducted on a weighted sample using women’s national sample weight. To handle the class imbalance in the data, SMOTE is used to resample the data to avoid overrepresentation of the majority class and underrepresentation of the minority class, where the class ‘delivery at home’ is a rare class and ‘delivery at any facility’ is the majority class in the nominal binary outcome variable, i.e., Place of delivery’. One of the pioneering works of Menardi and Torelli^48^ suggested how to handle class imbalances through the adoption of resampling techniques for binary data and proposed random oversampling examples (ROSE) as another efficient resampling technique. Studies by Zegeye et al.^5^ applied SMOTE while exploring similar problems with DHS data from sub-Saharan Africa. In the present study, we applied SMOTE oversampling technique to correct data imbalance. For example, previous study applying DHS dataset has shown the success of using oversampling to correct data imbalance as evident from the study of Khudri et al.^49^, on nutritional status prediction The application of resampling technique helps the current study to avoid prediction bias toward the majority class, poor learning of minority class under statistical learning process reducing evaluation bias and feature importance distortions. However, the study keeps in mind SMOTE’s limitation that synthetic samples can distort feature relationships and increase class overlap, risking overfitting and thereby affecting generalizability with inflated performance,k-fold cross-validation technique is used and variability across folds are reported to validate the performance.

The background profile of the respondents and the bivariate analysis followed by the chi2 test of independence are conducted under exploratory analytics. Under predictive modelling, 3 sets of models – without CHW factors, with CHW factors, with CHW factors and women’s perception about IPV are run. The 3 sets of models are run with nominal outcome variable having two categories (0=Facility birth’, 1=Home birth’). Comparison of 15 different models under ML is conducted with binary outcome variable following the study of Bwalya et al.^50^, and Li et al.^51^. Under predictive analytics, LRs are run on weighted data as classical models in three phases. Phase 1 includes only the meeting with the CHWs during the last 3 months preceding the survey, socioeconomic factors, lifestyle and access to service. Phase 2 controls the perception of women about intimate partner violence, and phase 3 includes interaction term which is visit of different CHWs with special focus on women who perceives intimate partner violence is justified. The Wald chi2 test estimates the explainability of the models, and ROC analysis estimates the area under the curve to compute the classification of true positive values compared with false positive values with respect to the three classes.

### The workflow diagram

- Step 1: Data file creation in STATA followed by Preprocessing (Cleaning, Encoding)
- Step 2: Handling Class Imbalance via SMOTE on weighted sample
- Step 3: Model building under classical predictive analytics followed by Training using ML algorithms
- Step 4: Model Evaluation using classification metrics
- Step 5: Explainability using SHAP

**Exhibit A:**
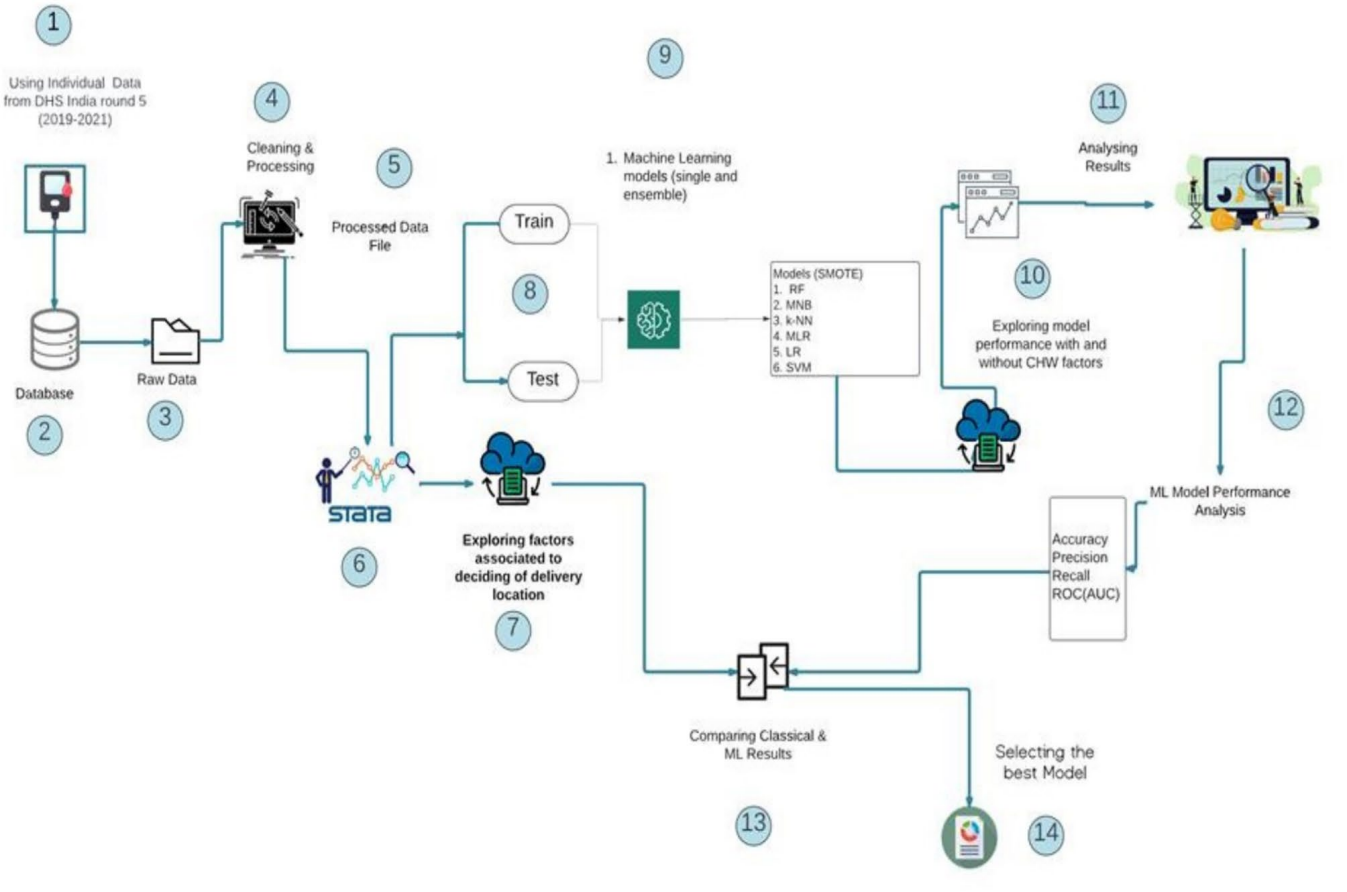
Workflow diagram

**Fig. 1:**
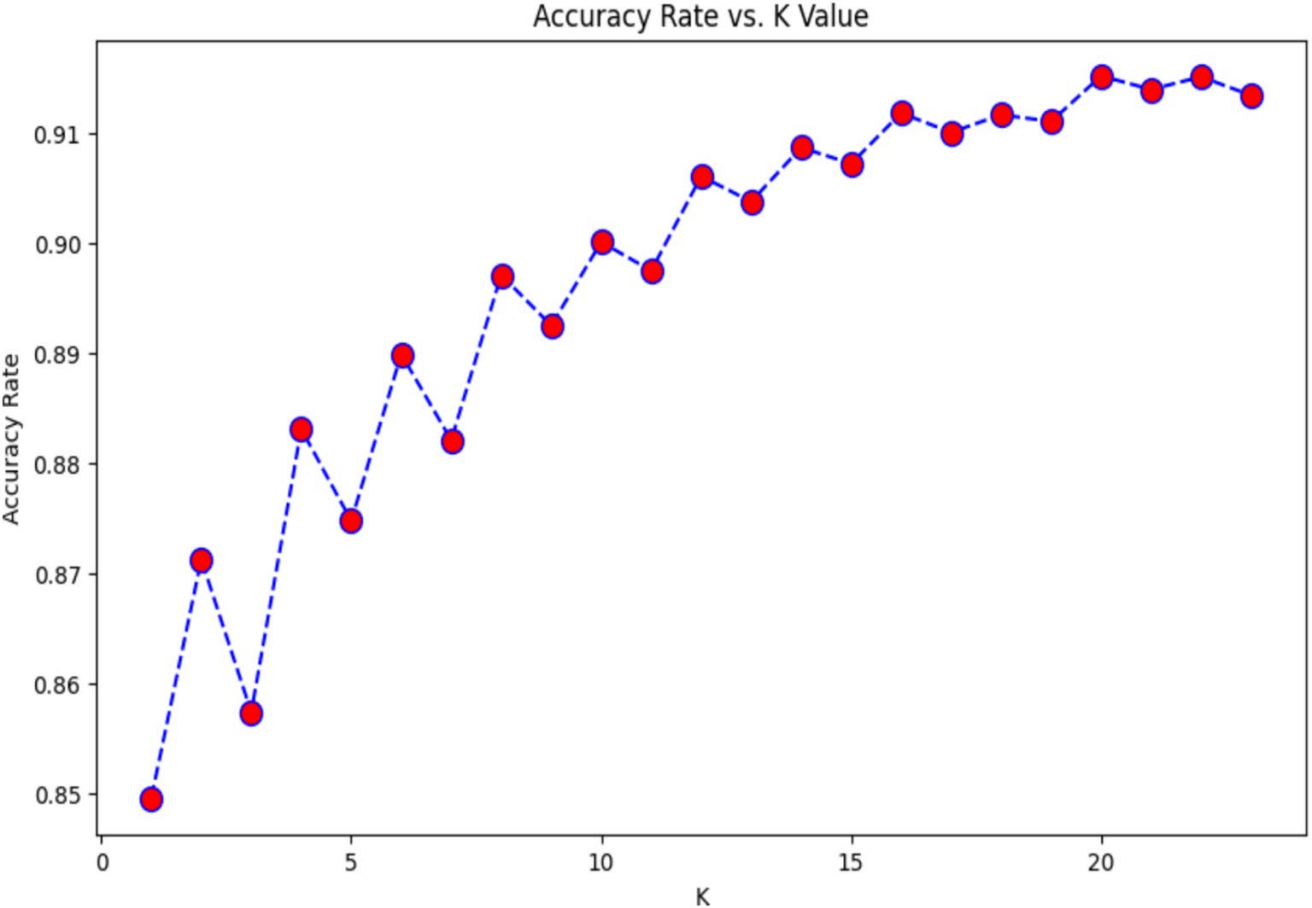
Accuracy vs. K-values.

Next, the machine learning models are run on weighted resampled data: to determine whether there is any change in the results of the evaluation metrics. RF, NB, k-NN, LR, and SVM supervised learning algorithms are used to predict the classification of test data (20%) objects based on the trained set (80%). The k-NN model was run for k = 15 nearest neighbours after estimating the accuracy curve for k=19 (after estimating the accuracy vs. k values as presented in Figure 1), and the best value of k was selected based on the highest predictability estimated via three distance metrics from the Lp Minkowski family (Euclidean, Manhattan) and inner product family (Cosine).

The accuracy, precision, recall, and ROC (AUC) values are estimated to assess the effectiveness of the five models to determine which is the best predictor in classifying the observations in the respective classes. All the features are categorical, numeric and labelled. The benefit of not transforming the features from one datatype to the other helps to estimate consistent similarity measures^52^. The complete workflow is explained in exhibit A. Finally, Shapely Additive exPlanations (SHAP) is run to identify the key contributing factors in the best select model (Figure 10). Furthermore, to train the models, the selected outcome/dependent variable taken for fitting the models is a binary variable, i.e., the ‘place of delivery of the child’ has two categories: ‘1: home birth’, ‘0: Facility birth’. Models are run incorporating seventeen features to conduct predictive analyses, as features are described in Table 1.

**Table 1:** Variable description.

Dependent variable	Type	Categories/Classes
Place of delivery	Nominal	Facility birth
		Home birth
**Feature**	**Type**	**Description of the feature**
**Contact with CHW related factors**		
Services/matters discussed	Nominal	• No service discussed • CHW discussed about Family planning, Immunization, ANC, delivery care, birth preparedness, complication readiness, PNC, disease prevention, Treatment of self, treatment of child
Frequency of meeting	Ordinal	• No discussion • Any 1 service discussed • Any 2 services discussed • 3 or more services discussed
Type of CHW met	Nominal	• No meet • Met ANM • Met ASHA • Met AWW
Met 1 or more types of CHWs	Nominal	• No meet • Met any 1 category of CHW • Met any 2 categories of CHW • Met any 3 categories of CHW • Met all the 4 categories of CHW
**Sociodemographic factors**		
Socioeconomic status	Ordinal	• Poorest • Poorer • Middle • Richer • Richest
Respondent’scurrent location	Nominal	• Urban • Rural
Education	Ordinal	• No education • Primary • Secondary • Higher
Ethnicity	Nominal	• Caste • Tribe • No caste/tribe
Water source for the household	Nominal	• Piped water • Tube well • Well • Spring • Rainwater • Bottled water
Sanitation	Ordered binary	• No access to flush toilet • Has access to flush toilet
Wife beating justified or not	Ordinal	• Wife beating is not justified • Wife beating is justified for any reason
Age at first pregnancy	Ordinal	• Teenage • Normal age
**Access to MCH service, maternal health and lifestyle factors**		
AntenatalCheckup (ANC)	Ordinal	• No ANC or < 4 ANC visits • 4 + ANC visits
Iron and Folic Acid (IFA)	Nominal	• Do not have/consume IFA • Consumes IFA Does not consume IFA though have it
Body Mass Index (BMI) status	Ordered	• Undernourished (< 18.5 kg/m2) • Normal (18.5 kg/m2—25 kg/m2) Overweight/obese (> 25 kg/m2)
Substance use	Ordered	• No use of substance Any one or more substance use
Status of anaemia	Ordered	• Severe • Moderate • Mild Not anaemic

The main feature of interest is the three different types of CHW – auxiliary nurse midwife (ANM), accredited social health activist (ASHA), and Anganwadi worker (AWW) visits with the respondents. The probabilistic analysis is conducted via machine learning algorithms by training the system followed by testing the predictability of the model based on training with 80% of the data and testing with 20% of the data to select the best model. The 5-fold cross validation technique is used to check any overfitting issue, comparing the fold specific achievements, Mean and Standard Deviation values followed by estimating the performance in a 20% held-out dataset. The metrics and evaluation estimates considered are the Euclidean, Manhattan and cosine distance metrics to find the best predictor distance metric in the case of the k-NN algorithm, recall, precision, accuracy, and receiver operating characteristic (AUC) curves are estimated to evaluate the performance of the models between the RF, NB, different k-NN, LR, and SVM models and the best machine learning model with the classical LR models. Stata 18.0 is used for data wrangling, conducting exploratory analysis and running classical regression models. To run machine learning models, python 3.11 is used.

## Results

### Background characteristics of the respondents

**Fig. 2:**
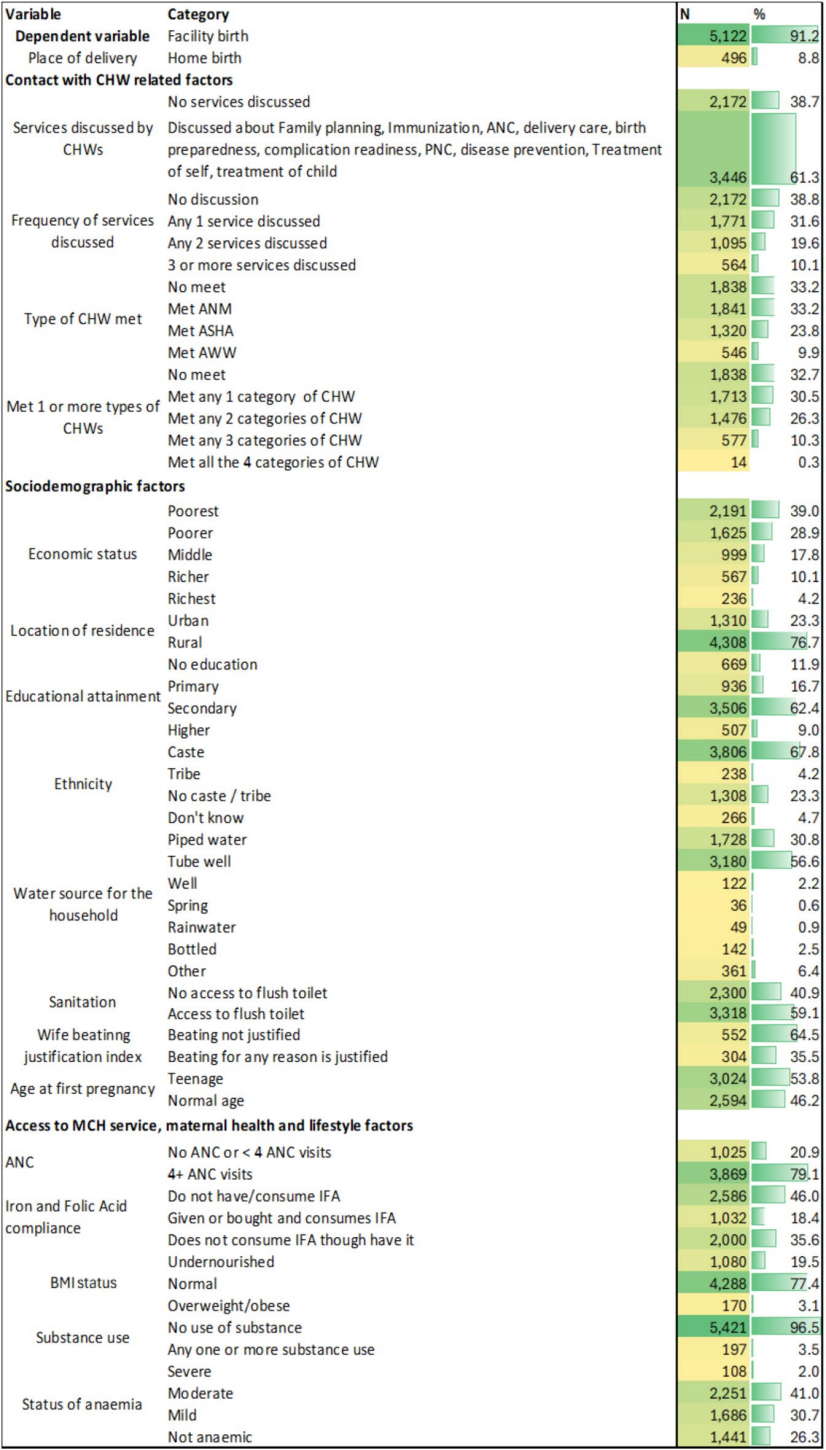
Background characteristics of the respondents.

Figure 2 represents the background characteristics of the respondents. The 5th NFHS round shows that 73% of the total deliveries took place in public facilities. A total of 38.7% of mothers reported that no discussion was held with any frontline workers in the past 3 months. (Among the total number of children whose birth history is collected, mothers are asked whether, in the past 3 months, they have received information on available MCH services, related care and management of illness, how to protect themselves from any exposure to health risks, and how to contact points of care services during any emergency—related to pregnancy or after the birth of a child—birth preparedness, complication readiness, postnatal care, medical treatment of self, and treatment of a sick child.) Among the different services, 30.2% of the mothers reported that CHWs discussed immunization services, and 18.6% received information about the family planning process and the services provided by the local health centres. Only 5% have received information regarding ANC, intrapartum and postpartum care services, and 7% are informed about care seeking during illness. A total of 31.6% of mothers received information about any 1 of these services, and 19.6% of the mothers reported that in the last 3 months, CHWs had discussed any 2 of the services. Only 10% of them were told approximately 3 or more services by CHWs in the recalled period. More than one third of the women did not meet any category of CHW in the last 3 months among the 4 categories – ANM, ASHA, MPW, or AWW. Among them, 30.5% met with any one category, 26.3% had discussions with any two categories of CHWs, 10% had discussion and information sharing by any 3 categories, and less than 1% had the opportunity to have information about services and knowledge sharing sessions on different health topics with all 4 categories of frontline workers. The variable considered as catalyst – the perception of women in support or against intimate partner violence – shows 35.5% women perceives intimate partner violence is justified.

The socioeconomic and demographic background of the respondents is as follows: 39% are poorest, 28.9% are poorer, 17.8% are middle class, 14.3% belong to rich families, 76.7% live in rural areas, 12% have no education, more than 60% are educated up to the secondary level, 72% belong to any caste or tribe, more than 50% drink water from tube wells, 59% have access to flush toilets, 49.4% are mothers of their first child, 35.1% have second order child, 15% have 3rd or higher order child, and 53.8% are mothers conceived at their teenage. Among the sample respondents, 22.6% were not normally nourished, 73.7% were anaemic of any degree of severity from severe (2%), moderate (41%) to mild (30.7%), 46% reported no IFA consumption and no IFA compliance was reported by 35.6%, almost 97% reported having no habit of substance use, and 79% had 4 or more ANC visits.

### Bivariate analysis

**Fig. 3:**
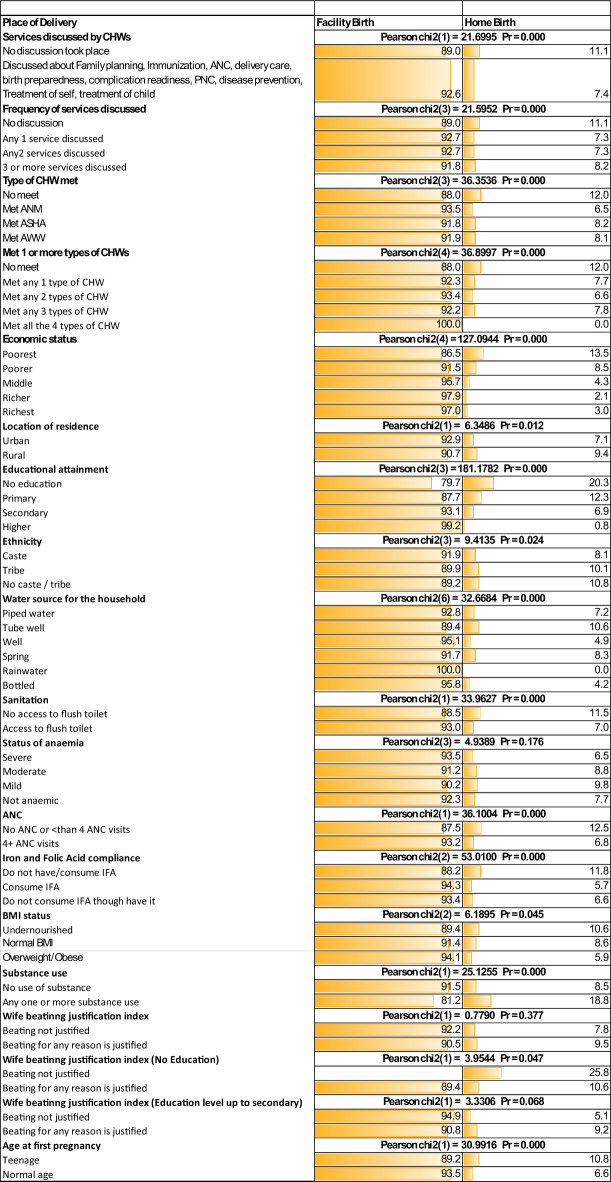
Bivariate analysis (chi2 test).

Bivariate analyses are conducted to test whether these background variables are associated with the outcome variable – the place of delivery of the child and presented in Figure 3. The null hypothesis of the chi2 test is that the variables have no association with the outcome variable. Among the mothers who received information on different MCH services and knowledge about preventive and curative care during and after pregnancy, 72% to 77% delivered at public hospitals (Pearson chi2 = 49.6456; p value = 0.000) and 93% delivered at any facility (Pearson chi2 = 21.6995; p value = 0.000). The percentage of home delivery is greater among mothers where no services are discussed, whereas the percentage of deliveries at hospitals is greater if the CHW has discussed one, two or more services and has enhanced their knowledge of healthy delivery and maternal and child healthcare (facility =74.9% - 77.3%, Pearson chi2 = 36.2423; p value = 0.000) and over 90% if considered birth at any facility (Pearson chi2 = 21.5952; p value = 0.000). This is evident when disaggregated by type of health worker (met ANM= 75.5%, 93.5%, met AWW= 78.2%, 91.9%, and met ASHA=74%, 91.8% delivered at a public facility, any facility respectively (Pearson chi2 = 36.3536; p value = 0.000) or meeting one (public hospital= 73.6%, any facility=92.3%), two (public hospital= 76.7%, any facility=93.4%), or three (public hospital= 77%, any facility=92.2%) different types of health workers (Pearson chi2 = 49.0603; p value = 0.000) and (Pearson chi2 = 36.8997; p value = 0.000).

Women from poor socioeconomic backgrounds (Poorest=86.5%, Richest=97%; Pearson chi2 = 127.0944; p value = 0.000), living in rural (90.7%) compared to urban (92.9%) residences (Pearson chi2 = 6.3486; p value = 0.012), not educated (79.7%) compared to educated up to the higher education (99.2%) level (Pearson chi2 = 181.1782; p value = 0.000), belong to any caste (91.9%) or tribal (89.9%) families (Pearson chi2 = 9.4135; p value = 0.024), undernourished (89.4%) compared to normal (94.1%), (Pearson chi2 = 6.1895; p value = 0.045) are significantly more likely to opt less for facility birth as visible from the observed data. Likewise, mothers who use more than one substance (18.8%, Pearson chi2 = 25.1255; p value = 0.000), do not have access to improved sanitation (11.5%, Pearson chi2 = 33.9627; p value = 0.000), had no or less than 4 antenatal visits (12.5%, Pearson chi2 = 36.1004; p value = 0.000), give birth before 20 years of age (10.8%, Pearson chi2 = 30.9916; p value = 0.000), women who perceive intimate partner violence is justified when they are not educated compared to educated (10.6%, Pearson chi2 = 3.3306; p value = 0.068) significantly more likely choose home delivery as evident from observed data.

### Predictive analysis

#### Classical regression models

Table 2 presents the results of classical regression models. The results of binary (BLR or simply LR) logistic regression models are run for three sets – without CHW, with CHW, with CHW + women’s perception about IPV, and reported here due to the fit of the LR models related to the research problem. Three binary logistic regression models are run to estimate the crude odds in model1 and adjusted odds ratios (aOR) to explore the odds of classifying home birth or facility birth where main independent variable considered is ‘participants met either ANM, or ASHA, or AWW’ compared to ‘no meet’ in model 2 and model 3. The discussion of different health problems, related preventive and curative services by either of them, their relationships with choosing home birth are analysed in Model1. Odds of delivering at home is significantly lower (OR=0.438; 95%) if had discussion with AWW compared to no visit by any of them. Model 1 shows visit of ANM or ASHA has no significant influence on the choice of facility birth. Surprisingly, when perception about intimate partner violence among women is controlled, results show there is 61.5%, 78%, and 95% lower odds of opting for home birth significantly if ANM visits, ASHA visits or AWW visits compared to no visit respectively. Model 3 includes an interaction term – type of CHW visit and women’s acceptance or non-acceptance of intimate partner violence. Women who accept intimate partner violence are significantly 3.49 times more likely to deliver at home. If ANM visits a woman who accepts intimate partner violence, her odds of home birth fall sharply, and they are significantly and 88% less likely at 90% level of significance. Therefore, visit of ANM plays significant role in reversing maternal or neonatal outcome when specific vulnerable groups are taken care of.

**Table 2:** The Binary logistic regression model.

	Model1	Model2	Model3
**No meeting (Ref.)**			
Met ANM	0.706	0.385*	0.910
Met ASHA	0.732	0.218**	0.469
Met AWW	0.438**	0.057***	0.000***
**Poorest (Ref.)**			
Poorer	0.678*	0.518	0.515
Middle	0.411***	0.056***	0.053***
Richer	0.212**	(omitted)	(omitted)
Richest	0.267**	(omitted)	(omitted)
**No education (Ref.)**			
Educated up to Primary	0.550**	0.208**	0.201**
Educated up to Secondary	0.427***	0.246**	0.226***
Have higher education	0.024***	(omitted)	(omitted)
**Urban place of residence (Ref.)**			
Rural place of residence	0.425***	0.348	0.301
**Caste (Ref.)**			
Tribe	0.783	0.642	0.589
No caste/tribe	1.470*	1.574	1.51
**Age at first pregnancy (teenage) (Ref.)**			
Age at first pregnancy (not teenage)	0.638**	13.618***	12.475***
Low BMI (< 18.5 kg/m2)			
Normal BMI (18.5 kg/m2—25 kg/m2)	0.931	0.347**	0.314**
Overweight/Obese (> 25 kg/m2)	0.433	1.503	1.821
**No anaemia (Ref.)**			
Mild anaemia	1.269	(omitted)	(omitted)
Moderate anaemia	1.026	6.434**	7.942***
Severe anaemia	0.336	3.533*	4.768**
**Do not use any substance (Ref.)**			
Use substance	1.659	(omitted)	(omitted)
**Consume iron and folic acid (Ref.)**			
No adherence to the consumption of iron and folic acid	0.935	0.409*	0.472
** < 4 antenatal care visits (Ref.)**			
4 or more antenatal care visits	0.741	0.662	0.698
**Have no access to safe water (Ref.)**			
Have access to safe water	0.986	0.848	0.834
**Have no access to flush toilet (Ref.)**			
Have access to flush toilet	0.727*	0.460*	0.441*
**Perceive wife beating is not justified (Ref.)**			
Perceive wife beating is justified		1.442	3.487*
ANM visits respondents who perceive wife beating is justified			0.117*
ASHA visits respondents who perceive wife beating is justified			0.152
AWW visits respondents who perceive wife beating is justified			5.96e + 06***
chi2	97.445	34.871	246.198
p	0.0000	0.0210	0.0000
Number of observations	2918	356	356

* p <.1; ** p <.05; *** p <.01.

**Fig. 4:**
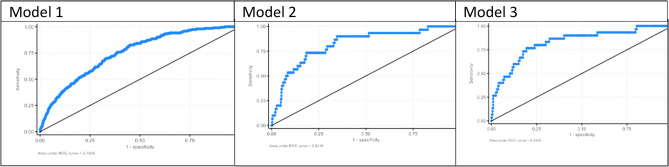
ROC analysis – Classical model.

The final model was run on 356 observations, Model 1 and Model 3 are significant at the 99% level and Model 2 is significant at 95% level. Moreover, Chi2 reflects the greater significance of Model 3 in comparison to Model 1 and Model 2 in rejecting the null hypothesis. In other words, the independent variables considered in the models predict the occurrence of delivery location, with true associations and not by chance. The variable ‘acceptance of intimate partner violence’ performs as a mediator and ANM’s visit works as moderating factor. The ROC analysis revealed that the overall performance of the classical model, as measured by the AUC, is the best for Model 3 (0.831) as found in Figure 4.

### ML models

The second phase of predictive analysis is more stochastic in nature, as the predictions from ML models are more complex and not easy to comprehend, such as straightforward regression analyses, since outputs vary in stochastic analysis, which is more stable and deterministic. The machine learning algorithms—random forest, naïve Bayes, k-nearest neighbors, logistic regression and support vector machine classifiers—are trained and tested to examine which model could achieve better performance with respect to classifying deliveries at home or public/private facilities evaluating different metrics.

Models are run in 3 phases, —without CHW- factors, with CHW factors, and with CHW factors + women’s perception about IPV, —to explore whether the predictive accuracy and overall performance vary with the inclusion of CHW factors and women’s perception on IPV on the resampled and weighted data.

**Fig. 5:**
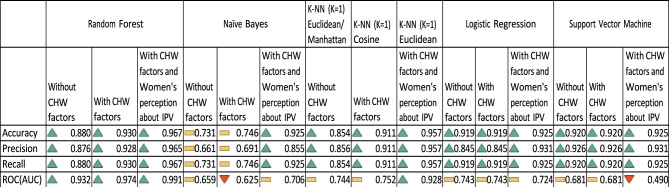
Performance comparison between different machine learning models.

Figure 5 presents the estimates (precision, sensitivity/recall) for the values k = 1for the three types of models. As per all three performance metric estimates, classes are better assigned to the first nearest neighbor under the cosine distance metric with CHW factors and the Lp Minkowski family of distance metrics without CHW factors, with CHW factors and women’s perception about IPV models. Therefore, among the final 15 k-NN models, k-NN with k = 1 under the Euclidean metric with CHW factors and women’s perception about IPV model is considered for model comparison with other single classifiers and the ensemble classifier RF.

**Fig. 6:**
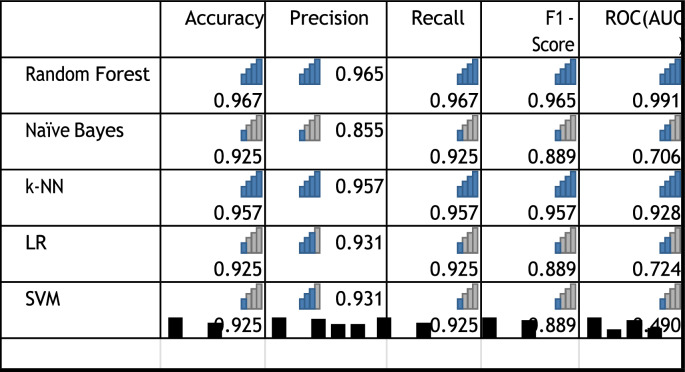
Performance comparison between machine learning models only for the models – with CHW factors and women’s perceptions about IPV.

Among the 15 ML models run on weighted resampled data, the RF with CHW factors and women’s perception about IPV was found to be the best model depicting the highest accuracy (96.7%), precision (96.5%), and recall (96.7%) compared to the random forest models with or without CHW variables and other ML models with or without CHW variables as well as with CHW factors and women’s perception about IPV. The accuracy (95.7%), precision (95.7%), and recall (95.7%) metric values from the k-NN with CHW variables and women’s perception about IPV is the second-best model. Figure 6 presents the model comparisons only related to the 3^rd^ set of factors (with CHW factors and women’s perception about IPV).

**Fig. 7:**
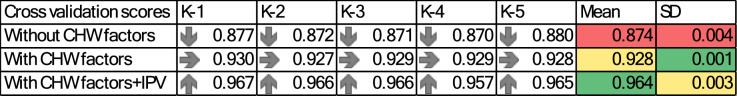
k-fold cross validation with fivefold (RF Model).

The 5-fold cross validation technique is used to check how the RF model performs in three scenarios (Figure 7). It is evident that the models are not overfitted. RF model without CHW factors has mean =0.874, SD=0.004 implying variations across folds are small with low risk of overfitting. RF model with CHW factors has mean =0.928, SD=0.001 indicates very good performance improvement with strong generalizability and no evidence of overfitting visible. RF model with CHW factors + IPV having mean=0.964 and SD=0.003 could raise the chance, however, SD is still low and stable performance across folds are evident. Therefore, considering the little risk of overfitting, testing is done on a held-out portion. The results show 96% accuracy with ROC(AUC)=0.950.

**Fig. 8:**
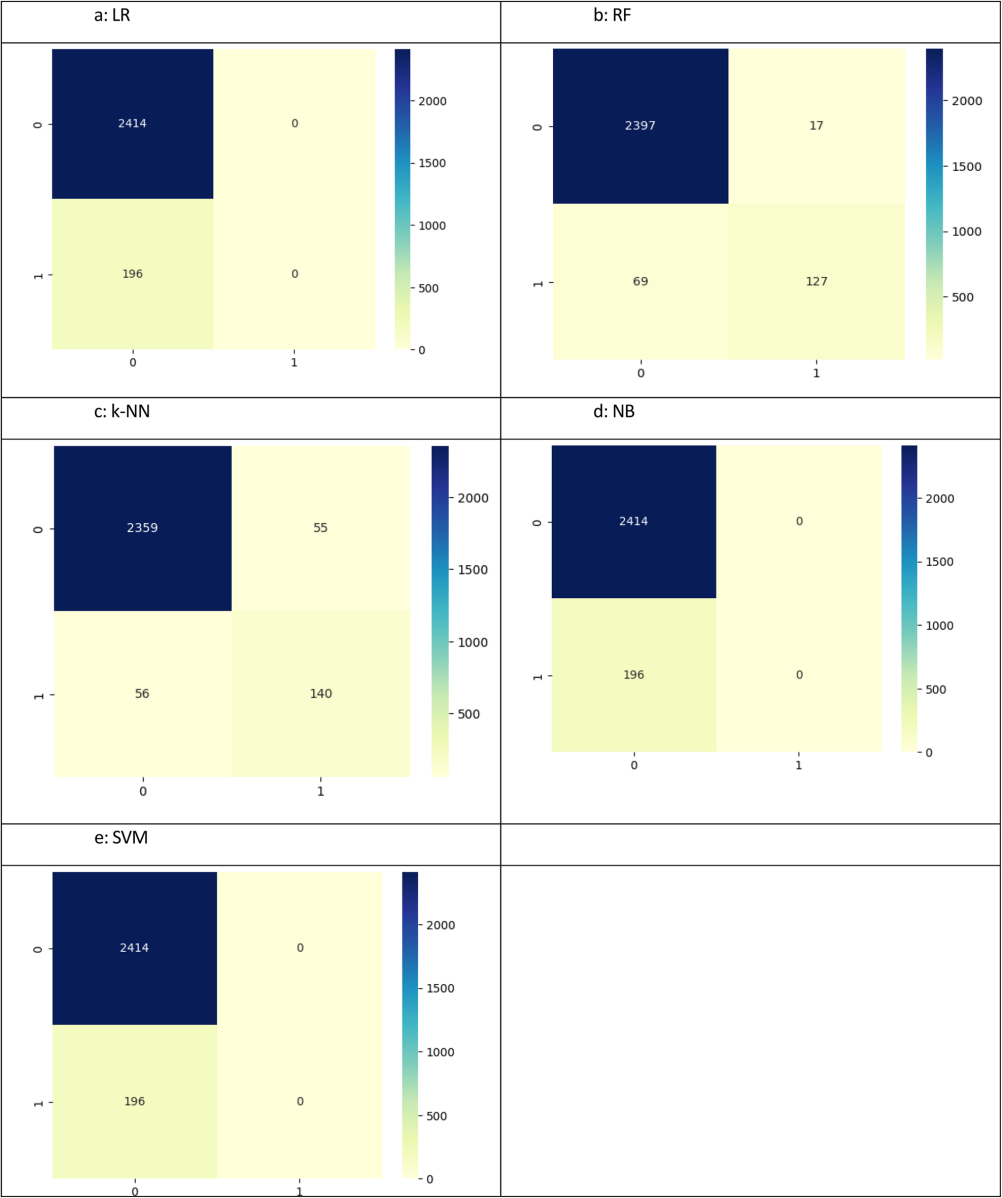
Confusion matrices.

Figure 8 depicts the confusion matrices of all the machine learning models representing how RF shown in Figure 8b made the classifications with the least errors (the most classification of TPs and TNs with least number of cases classified under false positives and negatives). Figure 8c is the second-best k-NN as it depicts second most classifications of true positives and negatives compared to false positives and negatives. The base mode LR (Figure 8a) and the other two NB (Figure 8d) and SVM (Figure 8e) performance reflect poor classification.

**Fig. 9:**
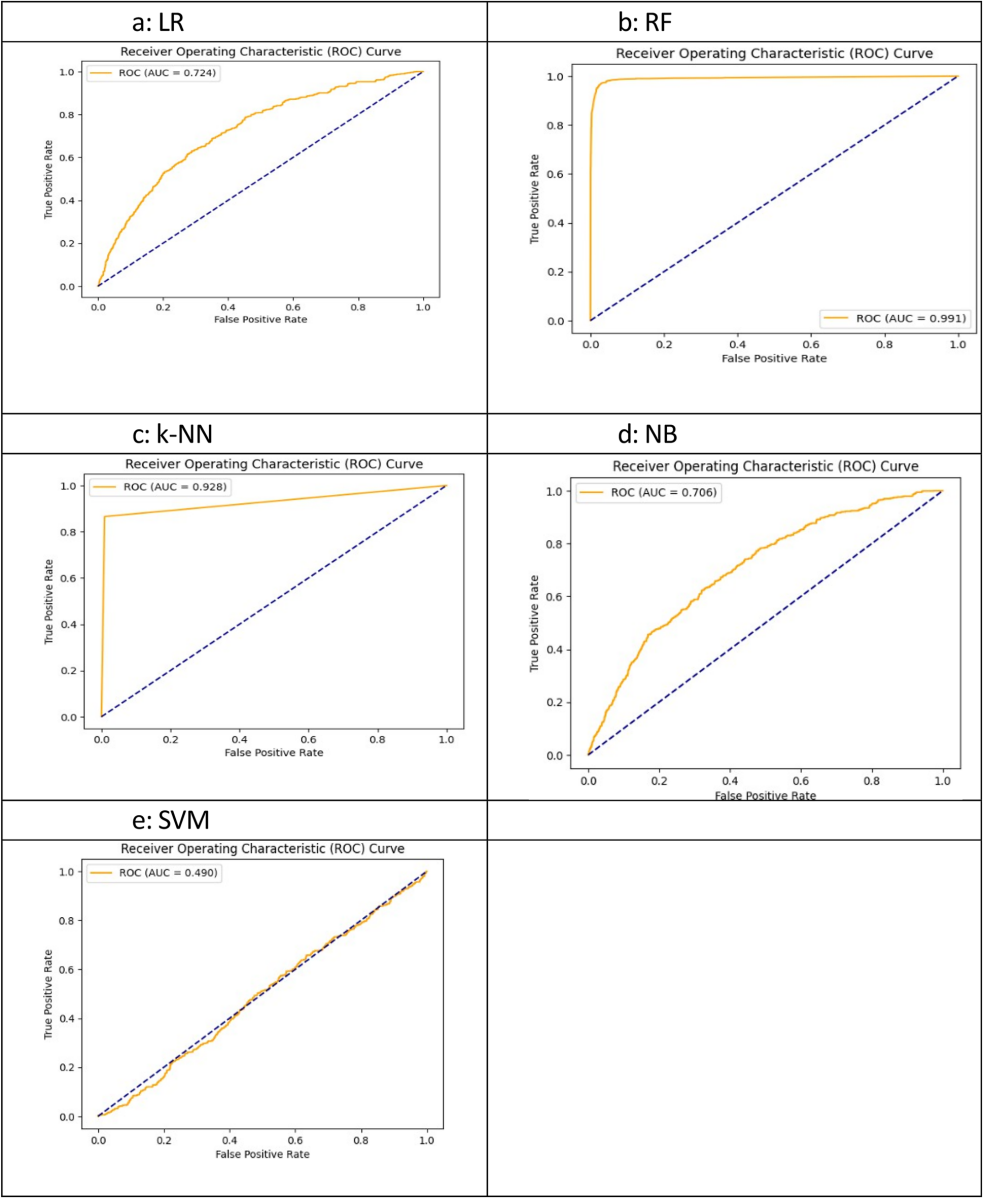
ROC analysis – machine learning models.

The results of the confusion matrices are reflected in a different way in the ROC(AUC) curves of the respective models in Figure 9. The area under the curve for the random forest in Figure 9b is the highest (0.991), depicting 99.1% ability to correctly classify the true positive values of the two classes with minimum type I error, whereas the k-NN being the second best shows an area under the curve equal to 0.928 in Figure 9c. The ROC(AUC) of the base model LR (Figure 9a) is 0.724 whereas NB (Figure 9d) and SVM (Figure 9e) cannot be used for prediction.

**Fig. 10:**
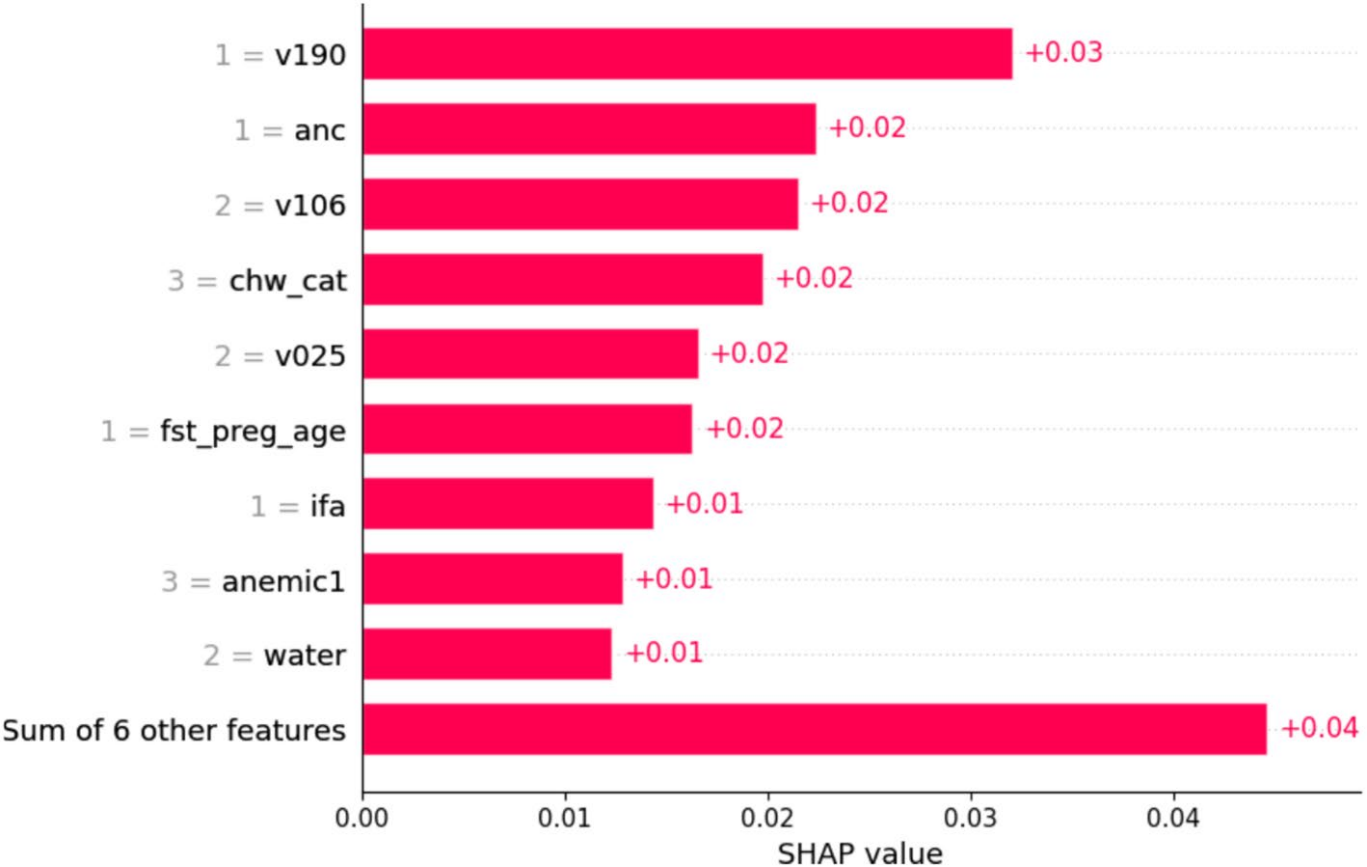
SHAP value.

SHAP values depict (Figure 10) major determinants are economic status, had 4 or more antenatal visits, level of education, type of CHW met for discussion, location of residence, age at first pregnancy. Women’s compliance with antenatal care, anaemia level, and access to proper living conditions moderately influence the outcome. The other 8 variables have marginal association independently. The model indicates women with less/no formal education, from rural area belonging to poor socioeconomic background who conceive early with less exposure to CHW meets need special attention to increase access to antenatal visits and CHW service discussions while targeting for increasing institutional delivery. This inference aligns with IPV related social dynamics and its associated influence on maternal healthcare access.

## Discussion

The current study aims to investigate the association between contact with CHWs and choice of delivery location. Classical regression analysis revealed a significant higher adjusted odds of choosing to deliver at home, the strength and direction of association is confounded by the type of CHW met. Secondly, the study runs different ML models to identify a better predictive model between 5 ML and 1 classical regression models to support the health management information system and automatically predict the association on a real-time basis and, at most, classify occurrences with higher accuracy, precision, and sensitivity. Five ML algorithms are applied, and 15 final models are compared after testing: RF, NB, k-NN, LR, and SVM. The models are trained and tested in three scenarios, — with and without meeting CHWs, and CHWs meeting women with special focus on them whose perception about IPV is collected. CHW meetings include discussion on self-care, newborn care, and maternal and child health services available in health centres, —and benefit from availing services, including the role of facilities, in ensuring better birth outcomes than home delivery of the child.

The RF classifier under the 3^rd^ scenario achieves the best classification report and explainability with the area under the curve. It is evident from ROC analysis that ML models performed better than classical models, indicating that the sensitivity with respect to 1-specificity was much greater for the RF model. The k-NN model under the Euclidean distance metric and the RF model have marginal differences in accuracy, precision and recall estimates. Nevertheless, RF is selected over k-NN (k=1) because the magnitude of the area under the curve is much greater in RF than in k-NN.

### Concordance with existing studies

Different studies have shown the application of RF in classifying health care-seeking outcomes in LMICs. A study by Gupta et al.^53^ used NB, RF, k-NN, SVM and LR MLR to classify the occurrence of cardiac arrest among newborns and proposed an ML model that is in line with the current study in relation to model selection. Several studies applying supervised earning models in disease prediction or health-seeking behaviour prediction have also concluded that RF is the best performing algorithm compared to other classifiers. Furthermore, a study by Yao et al.^54^ on the prediction of breast cancer diagnosis from 569 images revealed that the RF is the best performing model, with 96.3% accuracy, compared to the SVM (95.9%), which is in line with our study. However, the discordance lies in the type of data used for analysis. Nevertheless, Mani et al.^55^ applied k-NN, LR, NB, RF, and SVM to predict diabetes using a sample of 2280 records from demographic and clinical tests and reported that the area under the curve was greater for RF (0.803) than for other supervised algorithms (k-NN=0.721, LR=0.755, NB=0.762, SVM=0.749). Another study by Yang et al.^56^ used an SVM and an RF to predict diabetes from 9343 clinical and gene expression data and inferred that the RF is the best classifier, with 74% accuracy, whereas the accuracy of the SVM is 72%. Among similar studies, Juhola et al.^57^ reported that the RF classifier has the best accuracy (87.6%) compared to the k-NN (84.5%) and SVM (87.1%) classifiers in the prediction of heart disease from signal data. Toshniwal et al.^58^ reported similar results when the accuracies of NB (88.4%), RF (98.5%), and SVM (98.4%) were compared in an analysis of data from 47 electrocardiograms. Another study that predicted heart disease from 9637 demographic and hospital data reported that RF (89%) performed marginally better in terms of accuracy than did LR (88%) (Mansoor et al., 2017). Another study predicting the factors associated with the absence of mothers’ health-seeking behaviour for children with acute respiratory infections in sub-Saharan Africa on a weighted sample of 16832 records from DHS data reported that RF is the best model, with a 95.8% area under the curve, compared to DT (90.0%), XGB (80.2%), k-NN (75.8%), and LR (63.0%)^37^. In a previous study in which RF, LR, XGB, k-NN, and FCNN were used to predict high blood pressure, RF has shown the highest accuracy for Latin America and the Caribbean region and high performance, along with other good-performing models for other regions and for global-level analysis^59^.

Considering that the second-best predictability is achieved from the k-NN model, the results under the Lp Minkowski family of distance metrics in the ‘without meeting CHW’ scenario are found to be in line with other previous studies. Ali et al.^52^ used k-NN algorithms because of their well- known performance in classifying objects based on train-test sample distance estimation. They have taken Euclidean and Manhattan distances. However, in contrast to the current study finding where the Manhattan distance has shown better predictability with only one scenario at k=1, they found that it is true for k = 1, 5, and 7 when three different datasets are used. A similar finding is reflected in the study of Hu et al.^60^, who applied multiple distance metrics to estimate k-NN classifications on different medical datasets and reported that the Minkowsky and cosine methods yielded the worst predictors. In the current study, it is evident that the k-NN model is the second-best model in the ‘with CHW factors + IPV perception’ scenario and that the Euclidean metric is more effective in predicting the likelihood than the Manhattan or cosine distance metrics are.

The goal of the present study, i.e., improving health system performance applying AI, can be compared with that of previous studies where AI was used to enhance clinical diagnostic processes, e.g., deep k-NNs for image classification (Zhuan et al., 2020), or to strengthen public health service delivery via multiple machine learning models^5^. The current study used three parametric and three nonparametric models and a parametric model as the base model for predicting safe delivery locations, which is also in line with the findings of Fredriksson et al.^47^. It is also in line with the study by Zegeye et al.^5^, where they applied RF, DT, k-NN, EGB, AdaB, LR, ANN, and NB to a weighted sample of 299759 women from the DHS of sub-Saharan Africa to predict home delivery and identify its determinants. They also reported that RF (AUC=0.89) is the best model and that k-NN (AUC=0.85) is the second-best model.

Coming to the balancing technique used in the current study is the SMOTE oversampling technique to balance the imbalance between classes and avoid biased prediction in favour of the majority class. The class distribution after the weighted SMOTE was more equitable after the minority class samples were matched with the majority class samples. It helped to predict more accurately, reducing bias with robust performance, as found in Zegeye et al.^5^. In line with their study, higher accuracy and ROC (AUC) of the RF algorithms compared to any other algorithm are found in the weighted DHS data sample where SMOTE is used to balance the imbalance. Literature shows that data imbalance is mainly addressed following under sampling, oversampling and synthetic minority oversampling - are resampling techniques used to reduce compromises in the process of learning reflected in poor evaluation estimates^48^. As evident from the bivariate analysis, cardinality towards delivery at facilities is much greater than cardinality towards home delivery, which is a positive indicator of economic development. With that said, the home delivery class still contains positive values, and to bring perfectly equitable access to institutional delivery, higher accuracy in estimates is crucial. Otherwise, the pattern of classification with time will fail to support decisions toward efficient resource allocation, identifying people deprived of service in the direction of an optimistic scenario of increasing skewness favouring facility-based delivery with time. Conversely, the study is slightly affected by the limitation of SMOTE and reflects small chance of overfitting in the most crucial scenario from 5-fold cross validation (3^rd^ scenario). Nevertheless, hold-out validation on 20% unseen data reflects stable accuracy of 3^rd^ scenario RF model with little loss of generalizability. Given this context, different literary works who adopted efficient resampling techniques, among which SMOTE or ROSE were found to be more efficient than mere oversampling or under sampling techniques. Furthermore, studies by Zegeye et al.^5^ used SMOTE to address the influence of data imbalance in model evaluation while classifying and predicting health-seeking behaviour. The current study is in line with it. In the future, the next step of the current research will be to apply ROSE, as proposed by Lunardon et al.^61^and applied in Khudri et al.^49^,, instead of SMOTE or traditional resampling techniques to prevent any loss in generalizability mitigating the risk of overfitting.

### The wider LMIC context

Study by Huang et al.^62^ tested the applicability of a causal Bayesian network in predicting facility birth revealed quality of care as a crucial enabling factor. Another study by Fredriksson et al.^47^ made 68%–77% correct predictions of delivery location in the test sample as an outcome of a health worker programme in Zanzibar via a random forest model in line with the current study. The present study has shown higher accuracy of 93% with greater generalizability in the 2^nd^ scenario RF model indicating the significant role of CHW counselling. Additionally, the RF in the 3^rd^ scenario with small loss of generalizability with IPV added still can be considered as the best model with highest accuracy and ROC(AUC) given its contextual policy priority The community health worker programme in Zanzibar aimed to increase facility-based delivery through connecting pregnant women to facilities. estimated two parametric and two nonparametric models to make prediction. The current study workflow is grounded in the studies – e.g., Fredriksson et al.^47^, Zegeye et al.^5^ and applies three parametric models and three nonparametric models based on the type of the dependent variable, i.e., place of birth. Furthermore, the current study revealed better predictability and predictive accuracy of ML models compared to classical models not in line with Mboya et al.^35^ who found no significant difference between the two.

Another study involving an obstetrical cohort assessed the predictive accuracy of the super learner algorithm to identify preterm newborns in Lusaka, Zambia, using 4 maternal and 3 newborn features for gestational age modelling using NBS, LMP and birth weight^46^. In comparison, the current study incorporated 17 features, nevertheless, identified top 5 features using SHAP to prioritise focus area for health policy research.

The nonparametric machine learning algorithms are used to compute results that capture nonlinearities more flexibly, especially where nonlinearities are evident in relation to the type of CHW met and socioeconomic status or women’s autonomy is affected due to IPV is known and therefore easily identify the socially vulnerable women^47,63^. This is in line with the finding from the SHAP analysis which deduced the inference that identification of socially vulnerable women lacking perceived severity of IPV and needs to be connected to CHWs are associated with rural poverty, low educational attainment, less exposed to health service, and type of CHW allocated for service.. It is evident from multiple earlier studies that rural less educated women from poor socioeconomic background are victims of early marriage and early childbearing where IPV is a major moderator controlling their access to basic healthcare services^17,64,65^. On the other hand, a case-control study by Sahebi et al.^66^, has found that domestic violence is directly associated with severe maternal morbidity and deserves connection with health system which can only be possible through CHWs.

While analysing the SHAP results, Earlier literature shows optimistic results on CHW’s role in correcting the impact of IPV on women to improve their wellbeing^67^. The community-based intervention involving ASHA workers in a 4-phase implementation has resulted in reduced emotional and physical violence from husbands on women after 14 sessions and clinic-based interventions improved healthcare seeking^17,67,68^. Therefore, result-based identification of such women followed by connecting them to the health systems via CHWs can fade the evident association between the social barriers of access and birth at facility.

Furthermore, the inclusion of nonlinearities improved the predictability of the test sample with more accuracy, precision, and AUC which has optimistic application potential towards health programme strengthening. In addition, the present study inferred that the RF algorithm performed the best, which is advantageous over other algorithms because of its nonparametric nature, which reduces the misspecification risk, as found in the study of Fredriksson et al.^47^. The predictive accuracy of the RF model has become more reliable by reducing the impurity in the presence of high randomness while ranking features. RF is also more reliable due to its robustness to outliers and noise. The study by Zhang et al.^69^ explains how RF outperforms (AUC= 0.805) all the other selected algorithms – DT, LightGBM, SVM, LR, and XGboost.

The application of standard metrics to evaluate the classification performance is evident in studies with similar outcomes of interest. Like the present study, the study of Fredriksson et al.^47^ modelled skewed reporting of home delivery, computed the sensitivity and specificity values to assess the classification rates easing the performance evaluation technique. Furthermore, in the study of Rittenhouse et al.^46^, the super learner algorithm was found 94% accurate in identifying preterm births [ROC (AUC) = 0.980] saving time in the decision-making process. The ROC analysis revealed that after RF, k-NN was better in the 3^rd^ scenario, helping the decision on model alternatives. Similarly, it helps to decide on discarding the poor performing models where possibility of type I error was reflected in the low ROC (AUC) achievement after LR in the 3^rd^ scenario, in comparison to the 2^nd^ scenario. The higher the sensitivity is, the lower the tendency toward misclassification. In addition, a higher ROC (AUC) strengthens the classification ability, which is evident in the current study. Based on the above discussion it can be inferred that, the RF achieved ROC (AUC) of 0.991 with 5-fold cross validation and 0.950 with independent hold-out set, maintaining stable accuracy (96%) in the 3^rd^ scenario with IPV, Though it reflects some overfitting in the model it generalizes well to unseen data with discriminative ability sustained. This makes it ideal for detecting rare but significant patterns within IPV sample set.

### Novelty

The study utilises established ML algorithms to NFHS‑5 survey data. It does not bring any change in existing algorithms. The uniqueness lies in the application of advanced analytics to solve a crucial public health issue which is not done before for contextual policy decisions to the best of our knowledge. The study adds value by - incorporating CHW-contact types and IPV‑related perceptions into predictive analytics, comparing model performance across three policy‑relevant scenarios, and generated insights for programmatic interventions (e.g., prioritizing ASHA/ANM visits and customised CHW training to reach women who justify IPV). These contributions are contextually relevant and actionable to support public health decision-making instead of methodological innovations. It tests the applicability of conventional ML in the domain of ‘evidence-based healthcare practice’ targeting CHW programme design in India.

In line with the study of Shukla et al.^70^, the novel finding of the current study is identifying the immediate and underlying role of CHW factors in creating informed decisions even among disempowered women, followed by the proposal of the best ML algorithm. Finally, the best algorithm is proposed given the set of features to develop MCH decision support with the potential to conduct CHW performance analysis in real-time mode, design task-adjusted incentivization policies for efficient resource allocation.

### Future research directions

One of the most important aims of this study is to showcase how the data collected in the LMIC setting can be utilized to train ML models, and no dependency is required on HIC data to design a threshold, as also inferred in Sazawal et al.^71^. Future implementation would involve building MCH decision support using state-specific training data to improve the application of health management information systems (HMIS) in assessing CHWs’ ability to help pregnant women make informed choices of delivery locations in favour of public facilities.

Notably, in line with the study of Sazawal et al.^71^, the lower sensitivity with respect to the classification of home delivery might not reflect a weaker association, as the study did not consider any temporal variables. The lower number of object classifications might be due to the smaller number of home delivery cases evident in the sample. Therefore, further research based on a sample cohort is recommended to identify the factors and population parameters properly to explore each of the phases from pregnancy registration, antenatal visits, selection of delivery location, birth outcome, perinatal and neonatal health outcomes to control the rates of failed pregnancies, preterm birth, low birth weight birth, and neonatal mortality. Like other studies, predictive accuracy was estimated for the test models with 20% test data samples testing generalizability in held out set in the present work. It can be replicated for large population-based samples in other LMIC settings. However, there is a possibility of missing confounders, which should be complemented by primary research.

### Limitations

Despite direction-oriented performance, the current study has certain limitations. The study had a concern of data imbalance in the form of a lower cardinality of responses in the ‘Home birth’ category compared to the ‘facility birth’ categories in the outcome variable ‘place of delivery’. To address the data imbalance, the SMOTE resampling technique is run to balance the data between classes, which increases the likelihood of overfitting affecting generalizability. Along with it, the IPV subgroup (n = 356) represents a smaller sample limiting broader generalizability of the results. Consequently, though the RF model under 3^rd^ scenario reflecting the highest accuracy (96.7%), however, raises slight concerns about overfitting as evident from cross-validation results. It reflects the model is affected by a small risk of overfitting due to resampling using SMOTE, On the contrary, the 2^nd^ scenario RF model shows good accuracy with very good generalizability even after SMOTE as IPV variable (reducing the sample size) was absent. Nonetheless, the contribution of IPV cannot be ignored and 3^rd^ scenario possesses high policy relevance. Nevertheless, the hold-out validation confirms stable accuracy with no major loss in classification performance in the 3^rd^ scenario confirming its applicability. The extension of the current study will keep in mind applying multiple resampling techniques. Overcoming such limitations might be more time- and cost-efficient^48,49,61^.

## Conclusion

The current study shows ways to leverage machine learning algorithms over classical regression models to increase CHW led informed choice of facility birth giving special focus on women exposed to social vulnerabilities. The study proposes the best algorithm considering feature nonlinearities. It reflects the applicability of algorithms to help in formulating need-based CHW allocation and service delivery. A system of data collection, cloud-based storing, analysing to generate actionable insights and visualizing through real-time dashboard can be built to identify separate need-based clusters for intervention. The interactive results will help health centers allocate CHWs on a real-time basis in a periodic manner. It will create real-time performance-based incentive adjustments, reducing the monitoring time of the service delivery system. This will also help to identify the capacity building requirements for each CHW at the micro level connected to different programme component to correct diverse roots of unequal access.

Evidence from a nationwide survey reflects that although the rate of institutional delivery has increased from 75.2% to 91.7% in a period of 5 years, the rates of maternal and neonatal mortality are still high^7,11^. Knowledge about maternal and newborn care is crucial to ensure survival especially among poor and vulnerable women. Given the underlying role of CHWs’ home visits in conducting behaviour change communication over mere information sharing, the proposed workflow will help with timely access to the institutional care under trained attendants. Moreover, the present paper has adopted a novel approach of using AI to predict health workers’ role as a correlate—a smart shift in India’s primary healthcare model towards increasing the maternal and neonatal survival. Institutional delivery can be predicted with optimum social and maternal features to create a novel algorithm-driven decision support system for further predicting survival outcomes. A longitudinal study should be designed in continuation with the current work to identify the determinants of maternal and neonatal survival at each stage of pregnancy and childbirth—from pregnancy registration to 28 days of life—with greater accuracy and real- time data collection considering social, demographic, and geographical diversities.

**Fig. 11:**
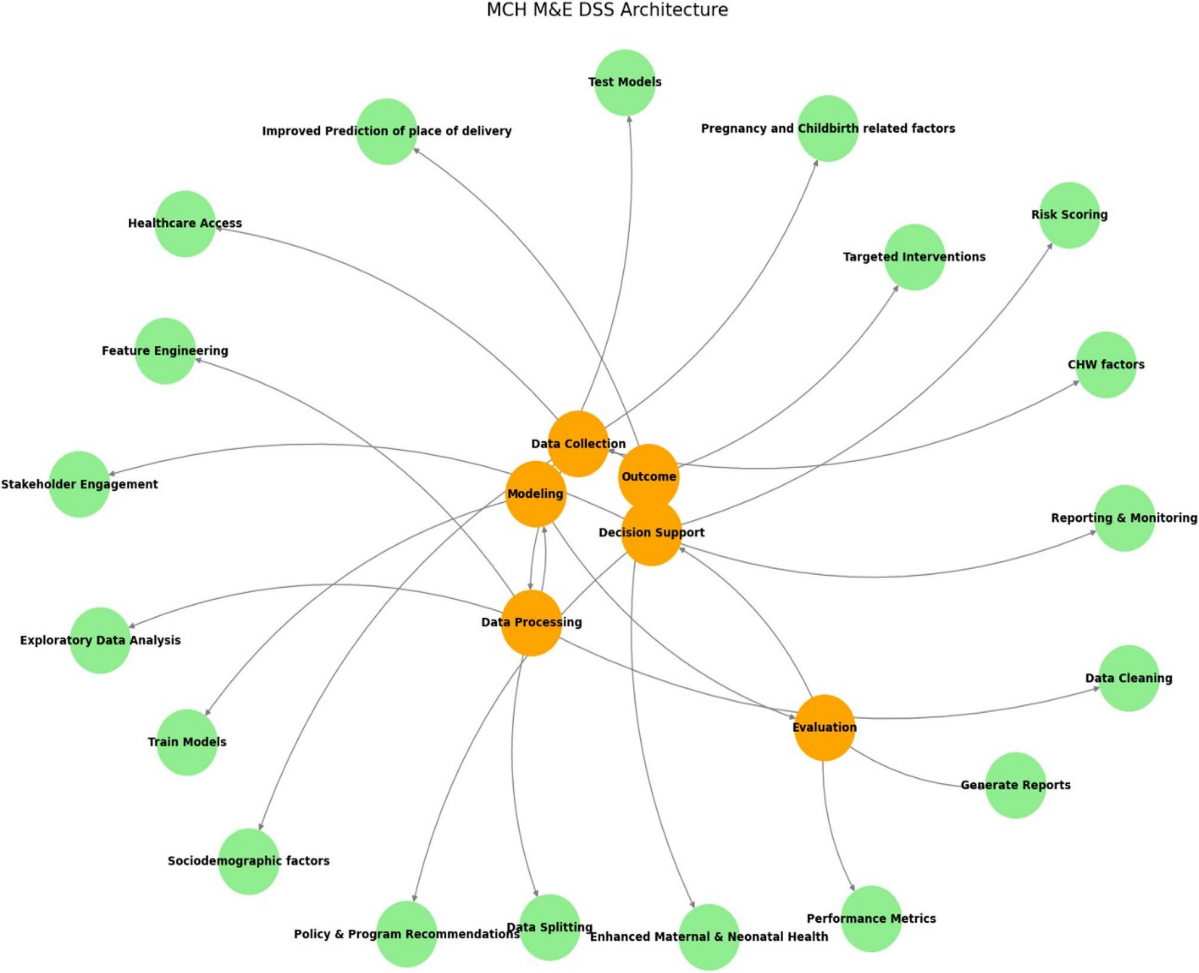
The proposed MCH monitoring and evaluation decision support system architecture.

Nevertheless, the proposed AI-driven digital health monitoring systems (Figure 11) need to be monitored on a regular basis. Ethical considerations in relation to data privacy, avoiding misuse, and ensuring fairness remain vital concerns in ML-based public health decision-making. Therefore, future research would explore the applicability of federated learning at the community level. The system might include triage assessment, meeting scheduling and home visits per schedule, storing in cloud-based database, connecting to the constructed algorithm to achieve the best context-driven predictive accuracy. The dashboard would reflect the pattern of likely delivery locations as women approach their expected delivery date. Any negative movement in the prediction trajectory can be identified per health worker, and initiatives at the individual level can help in making informed choices. At the population level, it would help reduce the rate of unsafe delivery and increase maternal and neonatal survival, dropping inequities in health status by equitable access.

### Evaluation metrics

**1. Accuracy** denotes to what extent the classifier classifies positives.

1 $$\text{Accuracy }= (\text{TP }+\text{ TN}) / (\text{TP }+\text{ FP }+\text{ FN }+\text{ TN})$$

**2. Precision** depicts the extent of true positive classification with respect to the total of true and false positives.

2 $$\text{Precision }=\text{ TP }/ (\text{TP }+\text{ FP})$$

**3. Recall** is also known as sensitivity, and it measures proportion of true positives correctly classified as true positives.

3 $$\text{Recall }=\text{ TP }/ (\text{TP }+\text{ FN})$$

**4. F1- score** estimates the ‘harmonic mean’ of two metrics—precision and recall balancing any imbalance giving higher weight to the lower value.

4 $$\text{F}1-\text{Score }= 2 * (\text{Precision }*\text{ Recall}) / (\text{Precision }+\text{ Recall})$$

where, how many cases are -

**TP (True Positives)** i.e. correctly predicted as positive, **TN (True Negatives)** i.e. correctly predicted as negative, **FP (False Positives)** i.e. incorrectly predicted as positive, **FN (False Negatives)** i.e. incorrectly predicted as negative.

**5. ROC(AUC):** The area under the ROC curve compares benefit over cost where the benefit of adopting the classifier is measured by sensitivity and the cost is measured by (1-specificity) where specificity measures the proportion of negative values correctly classified. One major benefit of accuracy, ROC(AUC) and the composite harmonic mean of precision and sensitivity is that these metrics are effective in measuring the performance of imbalanced data which is a protective measure to avoid any bias. Though our study tried to avoid imbalance through the application of resampling technique  , evaluation metrics chosen are helping to depict classification results with the least bias.

## Data Availability

Sources of Data The data that support the findings of this study are available from: [https://dhsprogram.com/data/available-datasets.cfm] The datasets used and/or analysed during the current study are available from the corresponding author upon reasonable request.
